# Targeted disruption of *Slc2a8* (GLUT8) reduces motility and mitochondrial potential of spermatozoa

**DOI:** 10.1080/09687680701855405

**Published:** 2008-04-21

**Authors:** Verena Gawlik, Stefan Schmidt, Andrea Scheepers, Gunther Wennemuth, Robert Augustin, Gerhard Aumüller, Markus Moser, Hadi Al-Hasani, Reinhart Kluge, Hans-Georg Joost, Annette Schürmann

**Affiliations:** ^1^ Department of Pharmacology, German Institute of Human Nutrition Potsdam-Rehbruecke, Germany; ^2^ Department of Anatomy and Cell Biology, Philipps-University, Marburg, Germany; ^3^ Department of Anatomy and Cell Biology, Saarland University, Homburg, Germany; ^4^ Max-Planck-Institute of Biochemistry, Martinsried, Germany

**Keywords:** GLUT8, glucose transport, sperm motility, mitochondrial membrane potential

## Abstract

GLUT8 is a class 3 sugar transport facilitator which is predominantly expressed in testis and also detected in brain, heart, skeletal muscle, adipose tissue, adrenal gland, and liver. Since its physiological function in these tissues is unknown, we generated a *Slc2a8* null mouse and characterized its phenotype. *Slc2a8* knockout mice appeared healthy and exhibited normal growth, body weight development and glycemic control, indicating that GLUT8 does not play a significant role for maintenance of whole body glucose homeostasis. However, analysis of the offspring distribution of heterozygous mating indicated a lower number of *Slc2a8* knockout offspring (30.5:47.3:22.1%, *Slc2a8^+/+^, Slc2a8^+/−^,* and *Slc2a8^−/−^* mice, respectively) resulting in a deviation (*p* = 0.0024) from the expected Mendelian distribution. This difference was associated with lower ATP levels, a reduced mitochondrial membrane potential and a significant reduction of sperm motility of the *Slc2a8* knockout in comparison to wild-type spermatozoa. In contrast, number and survival rate of spermatozoa were not altered. These data indicate that GLUT8 plays an important role in the energy metabolism of sperm cells.

## Introduction

Facilitated diffusion of glucose into mammalian cells along a concentration gradient is catalyzed by specific carriers (GLUT proteins). The GLUT family comprises 14 members and, according to sequence similarities, can be divided into three classes (Joost & Thorens 2001). GLUT8 belongs to the class 3 transporters and is an intrinsic membrane protein that catalyzes the facilitative transport of glucose and probably also fructose and galactose (Doege et al. 2000, Ibberson et al. 2000, Joost & Thorens 2001). A unique feature of class 3 transporters is the presence of endosomal targeting motifs that dictate their intracellular retention (Lisinski et al. 2001, Augustin et al. 2005, Schmidt et al. 2006). So far, translocation of class 3 transporters to the plasma membrane in response to a stimulus has only been demonstrated for GLUT8 in blasto-cysts (Carayannopoulos et al. 2000) and for the H^+^/myo-inositol transporter HMIT which belongs to the class 3 transporters due to its sequence similarities (Uldry et al. 2004).

GLUT8 is expressed predominantly in testis; smaller amounts of GLUT8 mRNA were detected in most other tissues including brain (cerebellum, brain stem, hippocampus, and hypothalamus), spleen, liver, and insulin-sensitive tissues such as heart, skeletal muscle, and adipose tissue. GLUT8 was detected in preimplantation embryos, in which it was reported to be essential for the development and survival of the embryo (Pinto et al. 2002). In testis, the GLUT8 protein was found to be associated with the acrosomal region of mature spermatozoa (Schürmann et al. 2002a), whereas others detected GLUT8 in differentiating spermatocytes of type 1 stage but not in mature spermatozoa (Ibberson et al. 2002). GLUT8 may also play a role in adipocyte metabolism because its expression increases markedly during fat cell differentiation, and is sensitive to prolonged hypoxia and glucose deprivation (Scheepers et al. 2001). However, in adipose cells, GLUT8 does not undergo insulin-stimulated translocation to the plasma membrane as described for the GLUT4 isoform (Lisinski et al. 2001). In liver, GLUT8 was detected in perivenous hepatocytes, indicating that GLUT8 might have a function in the regulation of glycolytic flux (Gorovits et al. 2003). Recombinant GLUT8 expressed in COS-7 cells exhibits glucose transport activity when reconstituted into lecithin liposomes (Doege et al. 2000). In addition, high-affinity glucose transport activity (*K*_m_ = 2 mM) was observed in *Xenopus* oocytes after injection of GLUT8 mRNA carrying a mutation of the amino-terminal dileucine motif (Ibberson et al. 2000). This activity was specifically inhibited by Dfructose and D-galactose, indicating that GLUT8 might be a multifunctional sugar transporter (Ibberson et al. 2000).

In order to analyze the specific role of the intracellular GLUT8 in glucose metabolism and function of sperm cells we generated a null mutant lacking *Slc2a8* and examined its phenotype with the focus on number and motility. While our study was in progress, it was described that targeted disruption of the *Slc2a8* gene in mice caused mild alterations in brain and heart, such as an increased proliferation of hippocampal cells and a slightly impaired transmission of the electrical wave through the atrium leading to a reduction in P-wave (Membrez et al. 2006). Here we report data demonstrating that GLUT8 is required for maintaining mitochondrial membrane potential and motility of spermatozoa. This study indicates that the intracellular compartment in which GLUT8 is expressed is needed for transport of metabolites required for energy production.

## Materials and Methods

### Antibody

A polyclonal antibody against two GLUT8-specific peptides corresponding to a sequence of the big intracellular loop (WGSEEGWEEPPVGAEG) and of the C-terminus (KGRTLEQVTAHFEGR) of mouse GLUT8 was raised in rabbit.

### Inactivation of the Slc2a8 gene

**Figure 1:**
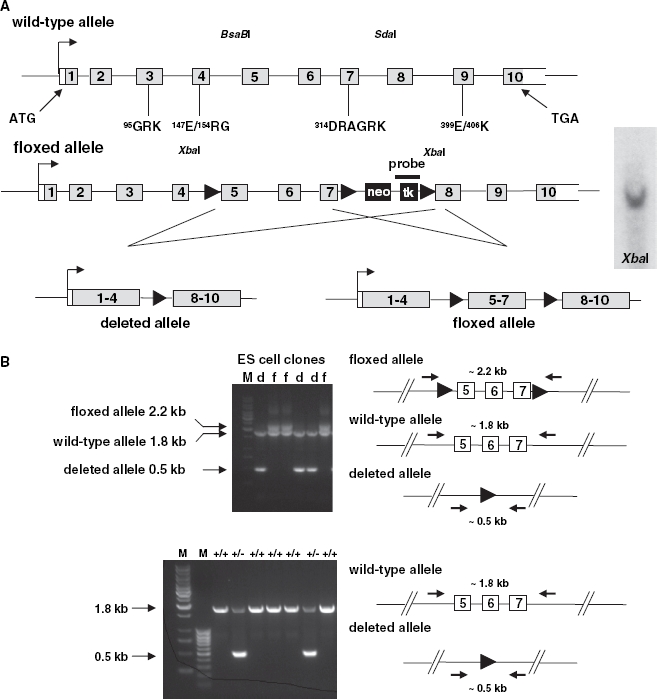

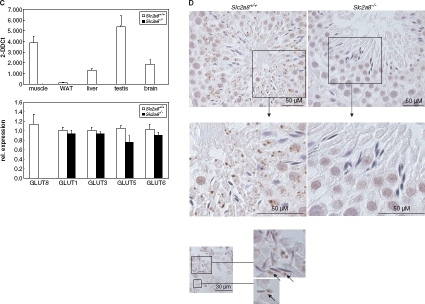
Generation of *Slc2a8*^flox^ allele and targeting of *Slc2a8*. (A) Targeting strategy which resulted in the introduction of loxP sites upstream of exons 5, downstream of exon 7 of *Slc2a8* and of a PGKneo/HSVtk cassette (Neo/tk) with a third loxP site. Verification of a single introduction of the targeting construct in the homologously recombined ES cell clone by Southern blot analysis as described in Materials and Methods (right panel). (B) After transient expression of Cre recombinase, the two types of ES cell clones carrying a deletion of exons 5–7 (d) or the floxed *Slc2a8* allele (f) were identified by PCR with specific primers matching to a 5′-flanking region of exon 5 and the 3′-flanking region of exon 7 (upper panel). Identification of *Slc2a8^+/+^, Slc2a8^+/−^*, and *Slc2a8^−/−^* littermates by PCR with specific primers as described under Materials and Methods (lower panel). (C) *Slc2a8* mRNA levels of 10 weeks old *Slc2a8^+/+^* and *Slc2a8^−/−^* littermates in muscle, white adipose tissue (WAT), liver, testis and brain were assayed by quantitative RT-PCR as described in Materials and Methods (upper panel). mRNA levels of GLUT8, GLUT1, GLUT3, GLUT5 and GLUT6 in testis of *Slc2a8^+/+^* and *Slc2a8^−/−^* males were assayed by qRT-PCR (lower panel). (D) Immunohistochemical detection of GLUT8 in seminiferous tubules. Sections of testis from *Slc2a8^+/+^* and *Slc2a8^−/−^* mice were fixed with paraformaldehyde and incubated with the anti-GLUT8 antiserum. The immunostaining was performed with peroxidase-conjugated secondary antibody as described in Materials and Methods. Sections were counterstained with Mayer’s hematoxylin (M, DNA ladders).

To generate a floxed *Slc2a8* allele we constructed a targeting vector (see Figure 1A). Exons 5 and 7 of *Slc2a8* were flanked with two loxP sites, and a PGKneo/HSVtk cassette (Neo/tk) with a third loxP site which was introduced downstream of the flanked exon 7. To verify a single introduction of the targeting construct in the homologously recombined ES cell clone, we blotted and hybridized the *Xba* III-digested ES cell DNA with the indicated probe giving a single band of 12.5 kb band for the wild type. Homologously recombined ES cell clones containing the targeted allele were transiently transfected with pCre to generate ES clones carrying a deleted allele and clones carrying the floxed *Slc2a8* allele. The two types of ES cell clones were identified by PCR with specific primers matching to a 5′-flanking region of exon 5 and the 3′-flanking region of exon 7. ES cells carrying the deleted allele were used for a morula aggregation. Blastocysts were then transferred into a pseudopregnant (day 2.5) female mouse. Male chimeric mice were mated with C57BL/6 females. Offspring carrying the transgene were backcrossed on to C57BL/6 3 times and subsequently intercrossed. The littermates of this intercross were used for the phenotypic characterization. Genotyping was performed by PCR (forward primer: 5′-CATCTTCTGTGCAGTCCATC-3′, reverse primer: 5′-GGTACCAAAGGCACTCATACTG-3′).

### Animals

The animals were housed in air conditioned rooms (temperature 20±2°C, relative moisture 50–60%) under a 12 h/12 h light/dark cycle. They were kept in accordance with the NIH guidelines for the care and use of laboratory animals, and all experiments were approved by the ethics committee of the Ministry of Agriculture, Nutrition and Forestry (State of Brandenburg, Germany).

### Southern blot analysis

DNA of ES cell clones was digested with *Xba*I, separated on a 0.7% agarose gel, and blotted onto a Hybond-N^+^ -nylon membrane (Amersham Pharmacia Biotech). To verify a homologous recombination of the targeting construct in the ES cell clones, a 522 bp fragment of the *Slc2a8*-gene, external to the targeting construct, was used for hybridization after labelling with [α^32^P]dCTP by using a random priming kit (Amersham Pharmacia Biotech). An 8.8 kb band of the wild-type allele and a 12.5 kb band of the floxed allele indicated a homologous recombination of the targeting construct (data not shown). For identification of a single introduction of the targeting construct in the homologous recombined ES cell clone, a 500 bp sequence that corresponds to the HSVtk-cassette was labelled and a single integration site of the targeting construct was indicated by a single band of 12.5 kb (Figure 1A).

### RNA preparation and first strand cDNA synthesis

RNA was extracted from different tissues and cDNA synthesis was performed as previously described (Buchmann et al. 2007). Quality of cDNA was controlled performing a PCR with murine GAPDH primers (forward: 5′-ACC ACA GTC CAT GCC ATC AC-3′; reverse, 5′-TCC CAC CAC CCT GTT GCT GTA-3′).

### Quantitative real-time PCR

Quantitative real-time PCR analysis (qRT-PCR) was performed with the Applied Biosystems 7300 Real-Time PCR System as described previously (Buchmann et al. 2007). The TaqMan gene expression assay (Mm00444635_g1) was used to detect the GLUT8-mRNA expression. The assay amplifies the region between exons 5 and 6, which is deleted in *Slc2a8^−/−^* mice. For the determination of other GLUT encoding genes, the following TaqMan gene expression assays were used: GLUT1 (Mm0044 1473_m1), GLUT3 (Mm00441483_m1), GLUT5 (Mm00600311_m1), GLUT6 (Mm00554217_m1). Data were normalized referring to Livak & Schmittgen (2001), whereas a b-actin expression assay (Mm00607939_si; Applied Biosystems) was used as endogenous control.

### Immunohistochemical detection of GLUT8

Paraffin sections of testis from *Slc2a8^+/+^* and *Slc2a8^−/−^* males were dewaxed in toluene and rehydrated, antigen demasking was performed by heat treatment (microwave, 2.5 min, 850 W) in Target Retrieval Solution (ChemMate^TM^, Dako Cytomation, Hamburg, Germany). Endogenous peroxidase was deactivated with 3% hydrogen peroxide for 10 min. Unspecific binding sites were blocked with DAKO antibody diluent for 30 min. The affinity purified polyclonal anti-GLUT8 antibody in a concentration of 5 μg/ml was applied overnight at 4°C in a humid chamber. Specific antibody binding was visualized by biotin-conjugated donkey anti-rabbit IgG (1:800; Dianova, Hamburg, Germany), for 30 min at 37°C, followed by incubation with a streptavidin-biotin-horseradish peroxidase complex (StreptAB-complex/POD) for 30 min, and diaminobenzidine as substrate according to the manufacturer’s specifications. The sections were dehydrated in graded ethanol and toluene and mounted in Histomount. Microscopic investigation and photo documentation were done with the combined light and fluorescence microscope ECLIPSE E-100 (Nikon, Düsseldorf, Germany) in combination with the video camera CCD-1300CB (Vosskühler, Osnabrück, Germany) and the Analysis System LUCIA G (Nikon, Düsseldorf, Germany).

### Preparation and characterization of Slc2a8^+/+^ and Slc2a8^−/−^ spermatozoa

For preparation of spermatozoa caudae epididymis and vasa deferentia were excised and rinsed with buffer as described (Schürmann et al. 2002b). Briefly rinsed spermatozoa were transferred to 1 ml medium supplemented with 5 mg of bovine serum albumin per ml and 15 mM NaHCO_3_, semen was allowed to exclude (15 min at 37°C, 5% CO_2_ from 3 to 5 small incisions). Cells were diluted to 4 ml and collected twice by sedimentation (400 *g*, 5 min). Sperm count and motility of the sperm cells were analyzed with a CASA (computer-assisted sperm analyzer, MTG, medeaLAB, Altdorf, Germany) as described by Krause (1995). Parameter settings were used as described by Wennemuth et al. (2003). For all assays sperm cells were kept in the indicated media not longer than 15 min in order to suppress capicitation (Wennemuth et al. 2003).

Cytoplasmic calcium levels of spermatozoa were assayed by loading the cells with 1 mM of the fluorescent calcium indicator Fluo3/acetoxymethyl (AM) ester (Gentaur; excitation: 506 nm, emission: 525nm recorded in FL-1, 530±30 nm band pass filter, FACSCalibur, BD) for 20 min at 37°C/5% CO_2_. After transport into the cells, AM hydrolysis occurs and, thereafter, the dye is trapped within the cell. Upon binding to calcium Fluo-3 exhibits a marked increase in fluorescence intensity (Perl et al. 2006). The mitochondrial calcium levels were estimated with 4 mM Rhod2/AM ester, which is compartmentalized into the mitochondria (Perl et al. 2006). Sperm cells were incubated with the dye for 20 min at 37°C/5% CO_2_ and emission was recorded in FL-2 (585±42 nm band pass filter).

The survival rate of spermatozoa was determined by staining with membrane-permanent nucleic acid dye 50 nM SYBR 14 according to manufacturer’s instructions (Molecular Probes) for 10 min at 37°C/ 5% CO_2_. Stained and unstained sperm were assayed by flow cytometry (excitation 488 nm, emission 520 nm allocated in the FL1 channel; FACSCalibur, BD).

Mitochondrial transmembrane potential (ΔΨm)of spermatozoa was likewise analysed by flow cytometry. 5,5′,6,6′-tetrachloro-1,1′,3,3′-tetraethylbenzimid-azolocarbocyanine iodide (JC-1) is a potential dependent J-aggregate forming lipophilic cation, which accumulates in mitochondria and indicates the membrane potential by an emission shift from green (527 nm) to red (590 nm, high transmembrane potential). Sperm where incubated with 2 μM JC-1 for 20 min at 37°C. JC1 fluorescence excited at 488 nm was collected with a 530±30 nm filter in the FL1 channel (green fluorescence) and a 585±42 nm filter in the FL2 channel (orange fluorescence). A preliminary staining with 50 μM carbonyl cyanide 3-chlorophenylhydrazone (CCCP) for 10 min was used as a positive control to disrupt the ΔΨm.

The ATP values of spermatozoa where determined by using the ATP Bioluminescense Assay Kit CLS II (Roche, Mannheim, Germany). The results were referred to the protein concentration of the samples.

### Serum parameters

Blood glucose levels, plasma insulin, triglycerides and free fatty acids were analyzed as previously described (Buchmann et al. 2007).

### Staining of acrosome

Sections of epididymal tubules of 12 weeks old *Slc2a8^+/+^* and *Slc2a8^−/−^* mice were incubated with 50 μg/ml FITC-conjugated *Pisum sativum* agglutinin (Sigma, St. Louis, MO, USA) for 30 min at 37°C in order to stain the acrosome as described earlier (Schürmann et al. 2002a).

### Glucose uptake

Uptake of [_14_C]-2-deoxyglucose by sperm of 12 weeks old *Slc2a8^+/+^* and *Slc2a8^−/−^* mice was determined as described (McLean et al. 1997). Briefly, isolated sperm were diluted to 0.5×10^6^sperm/ml with 50 mM TES, pH7.4, containing 130 mM NaCl and were centrifuged (700 *g*). Washed sperm were diluted in the above buffer containing 1 mM deoxy-D-glucose, and 2 μM CaCl_2_ and 1 μCi/μ1 [_14_C]-2-deoxyglucose and incubated for 15 min at 37°C. Glucose uptake was stopped by addition of a cold 0.1 M glucose solution and centrifugation at 12000 *g* for 5 min. Radioactivity of pellets was determined in a Beckman LS 6000LL scintillation counter.

### Statistical analysis

Statistical significance was determined by two-tailed Student’s *t*-test or by Pearsons’s chi-square test with 2 degrees of freedom for fertility mating. All values are the mean ±SEM. Statistical significance was set at *p* <0.05.

## Results

### Inactivation of GLUT8 by targeting the Slc2a8 gene in mice

For generation of GLUT8 knockout mice (*Slc2a8^−/−^* mice) we used the Cre/loxP recombination system which allows both the conventional deletion of the gene and the generation of tissue-specific null mutants. ES cells were transfected with the targeting construct (Figure 1A), and homologous recombinants were re-transfected with crerecombinase to generate deletion of the neo and tk cassettes (floxed allele) or of the full segments (deleted allele). For the generation of *Slc2a8^−/−^* mice, we performed a morula fusion with ES cells carrying the deleted allele as shown in Figure 1A (lower left panel). Male chimeric mice were mated with C57BL/6 females, and F1 progeny carrying the transgene were backcrossed 3 times on to the C57BL/6 background. Quantitative real-time PCR failed to detect full-length mRNA of Slc2a8 in all investigated tissues of *Slc2a8^−/−^* mice (Figure 1C, upper panel). Furthermore, no upregulation of GLUT1, GLUT3, GLUT5, or GLUT6 was observed in testis (Figure 1C, lower panel). Immunohistochemistry of testis sections performed with a specific anti-GLUT8 antibody raised against sequences corresponding to the intracellular loop 6 and the C-terminus of GLUT8 confirmed the absence of GLUT8 protein in testis of *Slc2a8^−/−^* mice while a positive staining of GLUT8 was detected in spermatocytes, spermatides, and mature spermatozoa of *Slc2a8^+/+^* littermates (Figure 1D). As shown in the lower panel of Figure 1D also in spermatozoa GLUT8 is located in an intracellular compartment rather than in the area of the acrosome as we have previously described (Schürmann et al. 2002a).

**Figure 2:**
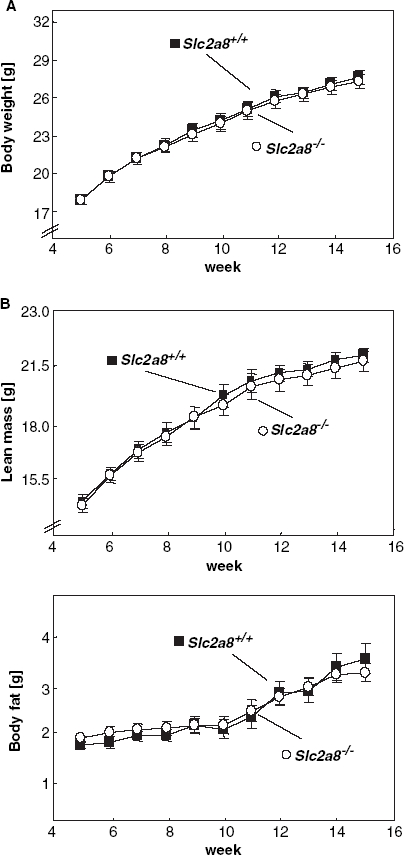
Characterization of body weight and body composition of wild-type and *Slc2a8^−/−^* mice. (A) Body weight, (B) lean mass (upper panel) fat mass (lower panel) development of male *Slc2a8^+/+^* and *Slc2a8^−/−^* mice (means±SEM from 36 *Slc2a8^+/+^* and 26 *Slc2a8^−/−^* mice).

*Slc2a8^−/−^* mice were viable with normal growth and no apparent abnormality. In sections from several tissues (liver, skeletal muscle, and adipose tissue) from *Slc2a8^−/−^* mice, no morphological or pathological abnormalities were observed (data not shown). Body weight development (Figure 2A) and body composition of *Slc2a8^+/+^* and *Slc2a8^−/−^* mice were identical (Figure 2B). In addition, no differences in serum glucose, triglyceride, free fatty acid or insulin levels were observed between fed wild-type and knockout mice (Table I). Due to these results we conclude that GLUT8 does not play an important role for the regulation of the energy balance and glucose homeostasis.

**Table I:** Comparison of plasma parameter in *Slc2a8^+/+^* and *Slc2a8^−/−^* males. Glucose, triacylglycerol (TG), free fatty acids (FFA) and insulin were measured in fed 12 to 15-week-old mice kept on standard diet. Data are shown as mean±SEM from 19–28 mice (as indicated in brackets).

	Glucose (mg/dl)	TG (mg/dl)	FFA (mg/dl)	Insulin (μg/I)
*Slc2a8^+/+^*	138.5±5.5 (*n* = 22)	72.1±3.6 (*n* = 23)	0.80±0.04 (*n* = 23)	3.3±0.4 (*n* = 28)
*Slc2a8^−/−^*	142.3±11.3 (*n* = 19)	70.0±6.4 (*n* = 19)	0.76±0.04 (*n* = 19)	3.9±0.6 (*n* = 25)

In the F2 progeny which was used for characterization of the phenotype, we obtained *Slc2a8^+/+^, Slc2a8^+/−^*, and *Slc2a8^−/−^* mice in a ratio of 30.5:47.3:22.1 (Table II). Since the number of homozygous knockout mice was significantly lower (*p* = 0.0024) than the expected 25%, breeding pairs of wild-type males and females, *Slc2a8^−/−^* males and females as well as *Slc2a8^−/−^* males and *Slc2a8^+/+^* females were formed, and mean litter size and number of pups born over a time period of 6 months were compared. No significant differences in the average litter size or the number of live pups were obtained between the indicated crossings (Table III), indicating that loss of GLUT8 does not cause a reduction in fecundity.

**Table II:** Numbers of offspring of *Slc2a8^+/+^, Slc2a8^+/−^*, and *Slc2a8^−/−^* genotypes obtained by crossing heterozygous mice.

Genotype	No. of offspring	%	Expected (%)
*Slc2a8^+/+^*	218 (113 males)	30.5	25
*Slc2a8^+/−^*	338 (186 males)	47.3	50
*Slc2a8^−/−^*	158 (89 males)	22.1	25

**Table III:** Test of fertility of *Slc2a8^−/−^* mice. *Slc2a8^+/+^* males and females (*n* = 7), *Slc2a8^−/−^* males and *Slc2a8^+/+^* females (*n* = 8), and *Slc2a8^−/−^* males and females (*n* = 9), were paired and mean number of litters and number of pups were recorded over a period of 6 months.

*Slc2a8* genotype			
		
Male	Female	Mean litter size	No. of pups
+/+	+/+	5.43±0.37	31.14±1.47
−/−	+/+	4.33±0.6	24.78±4.79
−/−	−/−	4.75±0.6	29.38±3.98

### Intracellular and mitochondrial calcium levels are not altered in *Slc2a8^−/−^* spermatozoa

**Figure 3:**
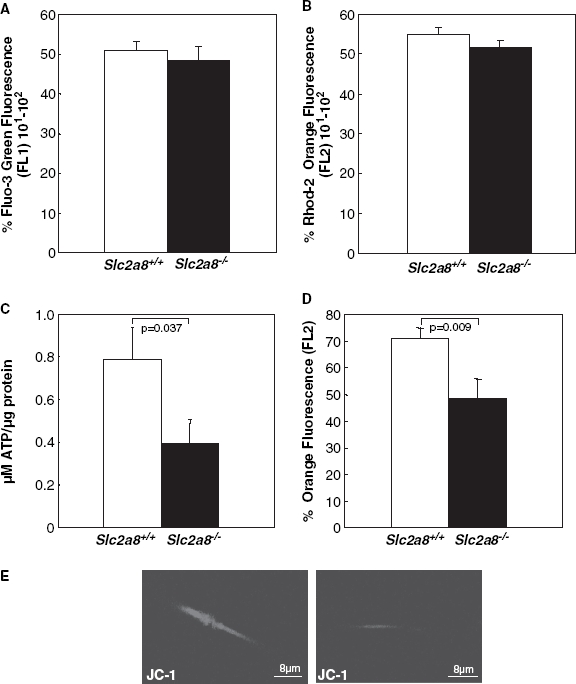
Detection of intracellular (A) and mitochondrial (B) calcium concentrations and assessment of (C, E) mitochondrial transmembrane potential and (D) ATP levels of spermatozoa from *Slc2a8^+/+^* and *Slc2a8^−/−^* littermates. Spermatozoa were isolated of *Slc2a8^+/+^* and *Slc2a8^−/−^* males at the age of 10–12 weeks. (A) Intracellular calcium levels were detected after incubation of spermatozoa with Fluo2/AM ester, (B) mitochondrial calcium levels with Rhod2/AM ester and analysis by flow cytometry. (C) ATP levels were measured as described in Materials and Methods. (D, E) Mitochondrial membrane potential was observed after staining of spermatozoa with JC1 (E) and subsequent flow cytometry analysis (D). Data present mean±SEM of 10 wild-type and 12 knockout mice. This Figure is reproduced in colour in *Molecular Membrane Biology* online.

The high expression levels of GLUT8 in testis and the slightly altered genotype distribution observed with heterozygous breeding pairs suggested an impaired function of mature spermatozoa in the absence of GLUT8. We therefore analyzed different parameters which have been associated with defective fertility, such as structural abnormalities (Escalier & David 1984, Lalouette et al. 1996), alterations of intracellular (Perl et al. 2006) or mitochondrial calcium concentrations (Okunade et al. 2004) or reduced mitochondrial membrane potential (Perl et al. 2006). Analysis of testis sections revealed no histopathology or indications that spermatogenesis was abnormal (Figure 1D). In addition, no differences in intracellular (Figure 3A) or mitochondrial Ca^2+^ concentration (Figure 3B) were detected with specific fluorescent calcium indicators in spermatozoa isolated from *Slc2a8^+/+^* and *Slc2a8^−/−^* mice.

### ATP levels and mitochondrial membrane potential are reduced in *Slc2a8^−/−^* spermatozoa

We next measured the ATP levels of isolated control and *Slc2a8^−/−^* spermatozoa and detected significantly (*p* = 0.037) reduced ATP levels of knockout sperm cells (0.39±0.11 μM/μg protein) in comparison to that of wild-type litter mates (0.78±0.15 μM/μg protein; Figure 3C). Beside glycolysis the mitochondrial phosphorylation plays crucial roles for the energy supply required for sperm motility (Turner 2006, Marchetti et al. 2002, 2004). Therefore, we analyzed the mitochondrial membrane potential (ΔΨm) of *Slc2a8^+/+^* and *Slc2a8^−/−^* spermatozoa by using the potentiometric fluorescent dye JC-1. As shown in Figure 3D ΔΨm was reduced by more than 20% in *Slc2a8^−/−^* spermatozoa. JC-1 staining as shown in Figure 3E detects mitochondria within the mid-piece of wild-type sperm. This staining was much lower in *Slc2a8^−/−^* spermatozoa. In addition, sperm viability was monitored by staining with Sybr 14 (Garner & Johnson 1995) but viability of *Slc2a8^+/+^* and *Slc2a8^−/−^* spermatozoa was not different (33.5±3.5 vs 31.9±2).

### Glucose uptake into Slc2a8^−/−^ sperm is not altered

In order to evaluate the effects of *Slc2a8* disruption on glucose transport into spermatozoa we measured uptake of labeled deoxy-glucose of isolated sperm cells from wild-type and *Slc2a8^−/−^* sperm. As expected from the intracellular localization of GLUT8 (Figure 1D) we did not detect differences in glucose transport between the two genotypes indicating that a reduced energy supply is not responsible for altered ATP levels of *Slc2a8^−/−^* sperm.

### Diminished motility of spermatozoa lacking GLUT8

In order to characterize the number and motility of *Slc2a8^+/+^* and *Slc2a8^−/−^* spermatozoa we excised epididymal tissue of *Slc2a8^+/+^* and *Slc2a8^−/−^* males at the age of 10–12 weeks, and analyzed them as described in Material and Methods. The sperm count of *Slc2a8^−/−^* males was not altered in comparison to that of *Slc2a8^+/+^* males (Figure 5B, left panel). In contrast, motility of *Slc2a8^−/−^* spermatozoa meaning the number of motile sperm cells as assessed with a computer-assisted sperm analyzer (CASA) was significantly (*p* = 3.3×10^−9^) reduced by about 50% compared to that of wild-type spermatozoa (Figure 5B, right panel). Within motile sperm no differences concerning the fast (≥25 μm/sec; 56.75±1.6 vs. 56.83±2.1%), slow (<25 μm/sec; 32.75±1.1 vs. 33.17±1.6%), and non-progressive local motility (between <5 μm/sec and > μm/ sec; 10.5±1.3 vs. 10.0±2.3%) was detected between *Slc2a8^+/+^* and *Slc2a8^−/−^* spermatozoa, respectively.

**Figure 4:**
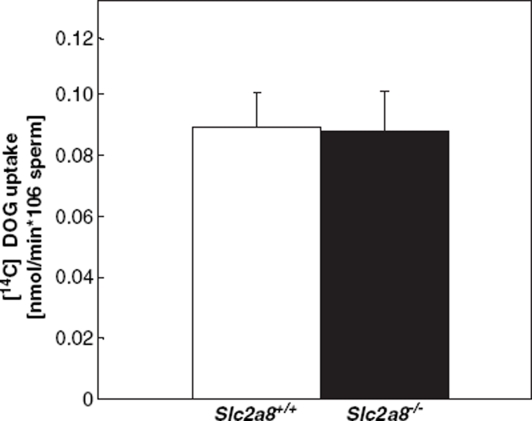
Uptake of [_14_C]-2deoxyglucose by sperm of 12 weeks old *Slc2a8^+/+^* and *Slc2a8^−/−^* mice. Each bar represents the mean and SE of observations from 6 mice.

**Figure 5:**
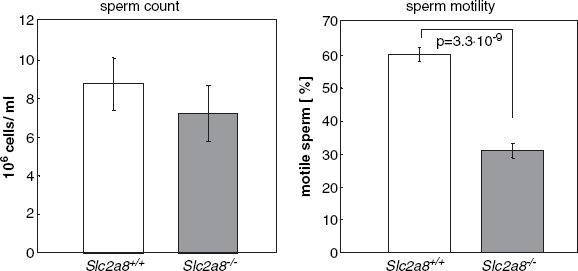
GLUT8 is required for normal sperm motility. Sperm count and sperm motility from *Slc2a8^+/+^* and *Slc2a8^−/−^* mice. Spermatozoa were isolated from epididymal tissues of 10–12 weeks old *Slc2a8^+/+^* and *Slc2a8^−/−^* mice and counted (left panel), and their mobility was analyzed as described in Materials and Methods (means±SEM from 6 mice of each genotype).

### Electron microscopic analysis of Slc2a8^+/+^ and Slc2a8^−/−^ testis

**Figure 6:**
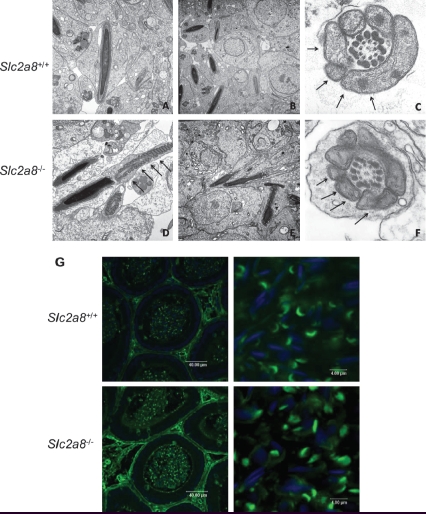
Electron microscopy of testis of *Slc2a8^+/+^* (A–C) and *Slc2a8^−/−^* mice (D–F) and analysis of cauda epididymal sperm with *Pisum sativum* agglutinine. (A) Early spermatides, (B, D, E) late spermatides, (C, F) cross section of a late spermatide, cut in the mid-piece region of the tail. Arrows depict the mitochondria (magnification approx. A and D × 18,000; B and E × 12,000; C and F × 40,000). (G) Sections of the cauda epididymis of *Slc2a8^+/+^* and *Slc2a8^−/−^* males at the age of 10–12 weeks were stained with *Pisum sativum* agglutinine and analyzed by confocal laser scanning microscopy. Nuclei were co-stained with DAPI.

To determine whether structural abnormalities are responsible for impaired motility of *Slc2a8^−/−^* sperm cells we examined testis of *Slc2a8^+/+^* and *Slc2a8^−/−^* mice by electron microscopy. As shown in Figure 6, in early and late spermatides of wild-type mice, normal development of acrosome and surrounding membranes was visible (Figure 6A, B) whereas development of mitochondria was not consistent within the epithelium of *Slc2a8^−/−^* testis. We observed a lack of condensation of mitochondria in some late spermatides (Figure 6E, F) but also late spermatides with normally developed mitochondria (Figure 6D).

In addition, we stained sections of cauda epididymus with *Pisum sativum* agglutinine which detects the acrosome. As shown in Figure 6G no differences between wild-type and knockout sperm could be observed indicating that the epididymal maturation of *Slc2a8^−/−^* sperm was not affected.

### Discussion

The present data show that the facilitative glucose transporter GLUT8 is required for maintenance of the mitochondrial transmembrane potential (ΔΨm) of spermatozoa. Ablation of GLUT8 reduces ATP levels of sperm and sperm motility which results in a slight but significant deviation of offspring from the Mendelian distribution of genotypes. However, sperm number and survival rate as well as glucose transport of sperm cells were unaffected by deletion of GLUT8. Our data give new evidence for an intracellular function of GLUT8, presumably the transfer of sugars or metabolites through membranes in order to maintain energy flow.

Motility and fertility of spermatozoa are regulated in a complex way. The role of glycolysis for ATP production is controversially discussed in the literature. An important study by Narisawa et al. (2002) has shown that under conditions of defective mitochondrial phosphorylation the sperm is still able to generate ATP and to fertilize. In addition, targeted deletion of sperm-specific GAPDH, an enzyme located in the fibrous sheath resulted in male sterility which was associated with severe aberrations in sperm motility (Miki et al. 2004). On the other hand, Ford (2006) demonstrated that glycolysis is not required to support normal sperm function. Inhibition of glycolysis by α-chlorohydrin did not alter mouse sperm motility. Therefore, beside energy provided by glycolysis sperm depend on production of reactive oxygen intermediates (Fisher & Aitken 1997) and maintenance of ΔΨm (Marchetti et al. 2002, 2004). Male mice lacking the enzyme transaldolase (TAL) which indirectly contributes to the generation of NADPH are sterile because spermatozoa lost ΔΨm and mitochondrial integrity (Perl et al. 2006). The effects on ΔΨm and ATP levels in *Slc2a8^−/−^* males did not lead to infertility; *Slc2a8* knockout sperm were able to fertilize *Slc2a8* wild-type oocytes. In addition, no differences in the number of litters or in the number of pups born after crossing *Slc2a8^−/−^* males with *Slc2a8^+/+^* females were observed in comparison to an intercross of *Slc2a8^+/+^* mice (Table III). However, the reduced number of motile *Slc2a8* knockout sperm resulted in a moderate but significant reduction of *Slc2a8^+/−^* (47.3 instead of 50%) and *Slc2a8^−/−^* (22.1 instead of 25%) offspring in the intercross of heterozygous mice when *Slc2a8* wild-type and *Slc2a8* knockout sperm competed for the oocytes (Table II). Altered mitochondria condensation as detected by ultrastructural analysis (Figure 6) might participate in the reduction of motility. We hypothesize that knockout sperm cells which exhibit defective mitochondrial condensation during development are those that are immobile after maturation. For the detection of mitochondrial membrane potiential and ATP levels all sperm cells were used including immotile sperm cells. Thereby reduced ΔΨm and ATP levels of the *Slc2a8^−/−^* spermatozoa were detected, indicating that the energy supply required for sperm motility was impaired. However, we did not detect differences in glucose uptake between *Slc2a8^+/+^* and *Slc2a8^−/−^* sperm (Figure 4). In addition, spermatozoa express the high-affinity glucose transporter GLUT3 at the cell surface (Haber et al. 1993, Burant & Davidson 1994). Therefore, it seems unlikely that deletion of GLUT8 which is primarily localized to intracellular membranes of all cell types (spermatocytes, spermatides, mature spermatozoa) impairs glucose uptake in Slc2a8 knockout cells. Rather, we propose that GLUT8 functions in a different, intracellular transport process which is required for mitochondrial function and generation of ATP.

The role of GLUT8 in the regulation of the energy homeostasis and the metabolism is so far poorly understood. Recently, Membrez et al. (2006) described a phenotypical characterization of *Slc2a8^−/−^* mice and observed normal embryonic and postnatal development, glucose homeostasis and response to mild stress, respectively. Here we confirmed the results of Membrez et al. (2006) who observed no differences in body weight between *Slc2a8^+/+^* and *Slc2a8^−/−^* mice kept on a standard chow diet. We therefore conclude that GLUT8 does not play a major role in the regulation of energy balance.

GLUT8 can be expressed in blastocysts and translocated to their surface in response to insulin (Carayannopoulos et al. 2000). In addition, suppression of GLUT8 expression by antisense mRNA caused increased apoptosis of blastocysts, suggesting that GLUT8 plays an essential role in early embryogenesis (Pinto et al. 2002). However, our data do not confirm this conclusion. Since mating of knockout mice produced viable, normally developing off-spring in numbers comparable to those of a wild-type intercross (Table III).

In summary, our data indicate that deletion of GLUT8 results in reduced sperm motility due to impaired mitochondrial function in spermatozoa, suggesting that GLUT8 is involved in the regulation of energy metabolism of sperm cells.

## Acknowledgements

This work was supported by the Deutsche Forschungsgemeinschaft (GK1208, FOR441, AU178/ 3-1). The skilful technical assistance of Brigitte Rischke and Elisabeth Meyer is gratefully acknowledged.
